# Room Temperature Ionic Liquids in Asymmetric Hetero-Ene Type Reactions: Improving Organocatalyst Performance at Lower Temperatures

**DOI:** 10.3390/molecules26020355

**Published:** 2021-01-12

**Authors:** Fabricio R. Bisogno, Rosario Fernández, Jose María Lassaletta, Gonzalo de Gonzalo

**Affiliations:** 1 Facultad de Ciencias Químicas, Instituto de Investigaciones en Físico-Química de Córdoba, Universidad Nacional de Córdoba, (INFIQC, CONICET-UNC), Haya de la Torre y Medina Allende, Ciudad Universitaria, Córdoba 5000, Argentina; fbisogno@fcq.unc.edu.ar; 2 Departamento de Química Orgánica, Universidad de Sevilla, c/Profesor García González 1, 41012 Sevilla, Spain; ffernan@us.es; 3 Instituto de Investigaciones Químicas (CSIC-US) and Centro de Innovación en Química Avanzada (ORFEO-CINQA), Avda. Américo Vespucio 49, 41092 Sevilla, Spain; jmlassa@iiq.csic.es

**Keywords:** organocatalysis, ionic liquids, ene-type reactions, tertiary alcohols, solvent engineering, asymmetric catalysis

## Abstract

Room temperature ionic liquids (RTILs) have been widely used as (co)solvents in several catalytic processes modifying, in most of the cases, the catalyst activity and/or the selectivity for the studied reactions. However, there are just a few examples of their use in hydrogen bonding organocatalysis. In this paper, we show the positive effect of a set of imidazole-based ionic liquids ([bmim]BF_4_ and [hmim]PF_6_) in the enantioselective addition of formaldehyde *tert*-butylhydrazone to prochiral α-keto esters catalyzed by a sugar-based chiral thiourea. Reactions performed in the presence of low percentages of RTILs led to an increase of the catalyst activity, thereby making possible to work at lower temperatures. Thus, the chiral *tert*-butyl azomethyl tertiary alcohols could be obtained with moderate to good conversions and higher enantioselectivities for most of the studied substrates when using up to 30 vol% of [hmim]PF_6_ as a cosolvent in processes performed in toluene.

## 1. Introduction

**Scheme 1:**
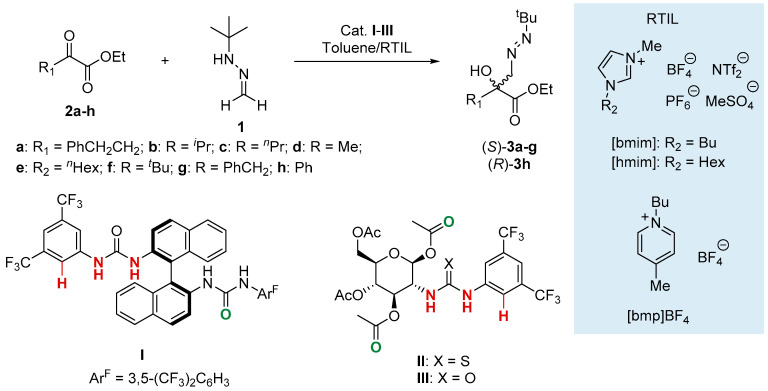
(Thio)urea-organocatalyzed preparation of optically active azomethyl alcohols (*S*)-**3a**–**h** employing catalysts **II** or **III** and preparation of compound (*R*)-**3h** using catalyst **I** in toluene containing different room temperature ionic liquids.

Room temperature ionic liquids (RTILs) are a class of organic salts that have found application in a number of contexts [1,2,3,4,5]. Among them, RTILs are currently used as solvents for different processes due to their unique properties, such as a high polarity, a poor (although tunable) coordination between anion and cation, and the fact that their structure can be easily modified. Moreover, it has been widely proved that ionic liquids may affect the outcome of different reactions, exerting beneficial effects in product recovery, reaction rate enhancement, and improved stereoselectivity [6,7,8]. For these reasons, the role of RTILs has been extensively studied as solvents or cosolvents in metal-catalyzed [9], biocatalyzed [10,11,12,13], and organocatalyzed reactions [14,15]. Regarding the latter, most of the examples reported so far refer to the use of ionic liquids in covalent organocatalysis. On the other hand, there are very few examples of their use in hydrogen-bonding catalysis using (thio)ureas or squaramides [16,17]. Very recently, a set of thiourea-based ionic liquids were used as catalysts for the cycloaddition of CO_2_ to epoxides. These imidazolium-based thioureas showed an improved activity due to the coexistence of the imidazole and the thiourea group as active moieties in the molecule [18,19]. 

(Hetero)ene reactions comprise pericyclic 6-electron processes in which an (hetero)unsaturated compound bearing an allylic hydrogen (the “ene”) reacts with an electrophilic (hetero)unsaturated partner (the “enophile”) to give rise to the formation of two σ-bonds and migration of the π-bond [20,21,22]. When carbonyl compounds are used as the enophile, these reactions provide valuable functionalized alcohols. Asymmetric carbonyl-ene reactions have been mostly carried out through metal-catalysis [23,24,25,26]. Nevertheless, in the last years, organocatalytic versions have been successfully developed [27,28]. In this context, formaldehyde *tert*-butylhydrazone (FTBH, **1**) has been successfully tested in selective organocatalyzed hetero-carbonyl-ene reactions with carbonyl compounds (α-keto esters [29,30], isatins [31], α-keto phosphonates [32], simple aldehydes [33], and fluorinated ketones [34]) to obtain functionalized chiral alcohols, valuable precursors for the synthesis of α -hydroxy aldehydes, 1,2-diols, or 1,2-aminoalcohols [29,30]. Reactions of aromatic α-keto esters **2** [(R = (hetero)aryl] were effectively catalyzed by BINAM-derived bis-urea (**I**) [BINAM = (*S*)-2,2′-bis(diphenylphosphinoamino)-1,1´-binaphthyl], leading to the corresponding *tert*-butyl diazenyl carbinol (*R*)-**3h** in both excellent yields and enantioselectivities (Scheme 1) [30]. Regarding the addition to aliphatic α-keto esters (*S*)-**2a**–**g**, only moderate enantioselectivities were achieved, using in this case carbohydrate-based thiourea **II** as the catalyst [29]. Thus, the addition of **1** to compounds **2a**–**g** catalyzed by **II** was carried out in toluene at −15 °C in order to obtain good conversions. Reactions performed at lower temperatures required long times to achieve just poor to moderate conversions [29]. Considering that RTILs can be efficiently applied to related carbonyl-ene metal-catalyzed processes [35,36], the organocatalyzed hetero-carbonyl-ene reaction of aliphatic α-keto esters was chosen as a suitable platform to explore the effect of a set of RTILs as cosolvents or additives in the catalyst performance.

## 2. Results and Discussion

Initial experiments were performed to analyze the effect of small amounts (10 vol%) of different RTILs in toluene in the addition of hydrazone **1** to ethyl 2-oxo-4-phenylbutyrate (**2a**) catalyzed by **II** (10 mol%), as shown in Table 1. This amount of RTIL was deliberately chosen for the first set of experiments to ensure a 1:1 ratio of catalyst/RTIL, assuming that at least one molecule of RTIL interacts with one molecule of catalyst. In the absence of RTIL, the azomethyl alcohol (*S*)-**3a** was obtained with 68% conversion, measured by ^1^H-NMR and 64% ee after 16 h (entry 1). In order to compare the effect of the RTIL on the catalyst activity independent of the reaction time, we also determined the catalyst turnover frequency (TOF, h^−1^), which reached a value of 0.43 h^−1^ in this reference experiment. When employing different imidazole-based ionic liquids (entries 2–6), the reaction was accelerated. Thus, similar conversions were achieved in shorter reaction times (7 h). TOF values under these reaction conditions were 2 to 3 times higher than in pure toluene. The use of [bmim]BF_4_, [bmim]PF_6_, and [hmim]PF_6_ led to highest conversions of the α-keto ester, while the optical purities were the same as those achieved in toluene for [bmim]BF_4_ (entry 3, 63% ee) and [hmim]PF_6_ (entry 5, 65% ee). Imidazole-based ionic liquids containing NTf_2_ and especially MeSO_4_ as anions had a negative effect in the system stereoselectivity. This phenomenon can be ascribed to interferences in the proposed recognition mode, as compared to BF_4_^–^ and PF_6_^–^-containing imidazolium-based RTILs. The reaction was also performed with 10 vol% of the pyridine-based ionic liquid [bmp]BF_4_ (entry 7). After 7 h, a 45% conversion was achieved, showing a worse performance than the imidazole-based ionic liquids, but still higher TOF values than the reference reaction were recorded (TOF 0.64 vs. 0.43 h^−1^, respectively). Molar fraction (*X*_RTIL_) of ionic liquids were calculated for a 10 vol% volumetric ratio. As can be observed in Table 1, *X*_RTIL_ for each cosolvent depended on the structure of the cation and the anion molecular weight and varied from 3.5 to 5.9%. Unfortunately, no clear trend between the molar fraction percent and the TOF could be established. 

In view of the higher activity found at −15 °C using a 10 vol% of certain imidazole-based ionic liquids, the temperature was lowered to −30 °C in the presence of [bmim]BF_4_ or [hmim]PF_6_, aiming to reach higher enantiomeric excesses of the azomethyl alcohol (*S*)-**3a** (entries 9 and 10) at lower temperatures. After 22 h, (*S*)-product was obtained in both cases with conversions of 83% and 89%, respectively, and optical purities around 70% ee, while the reaction in pure toluene occurred with a 29% conversion and the same optical purity (entry 8). At this temperature, the TOF for this reaction when using [hmim]PF_6_ was 3 times higher than in absence of RTIL. Temperature was further lowered to −45 °C. Under these conditions, the organocatalyzed process in pure toluene led to a 21% conversion of (*S*)-**3a** after 24 h, reaching a slightly better 71% ee (entry 11), while the addition of the RTILs increased the conversions up to 70% in the same reaction times (entries 12 and 14), keeping the enantiomeric excess of the (*S*)-alcohol around 75%. This means a 3-times increase in TOF values. The reaction was also tested employing as a catalyst urea **III**, an analogue to thiourea **II** (entries 13 and 15). For both ionic liquids, conversions and selectivities in the reactions catalyzed by **III** were slightly lower when compared with **II**. The organocatalyzed reaction in toluene alone and containing 10 vol% [hmim]PF_6_ was also carried at –60 °C (entries 16 and 17), but at this temperature the final product (*S*)-**3a** was obtained with no improvement in optical purity as at –45 °C. The reaction was slowed down to a significant extent in presence of 10 vol% [hmim]PF_6_ (34% conversion after 72 h, entry 17). However, the reactivity virtually doubled in the presence of the ionic liquid (TOF 0.03 vs. 0.06 h^−1^, in absence and presence of RTIL, respectively). 

**Figure 1:**
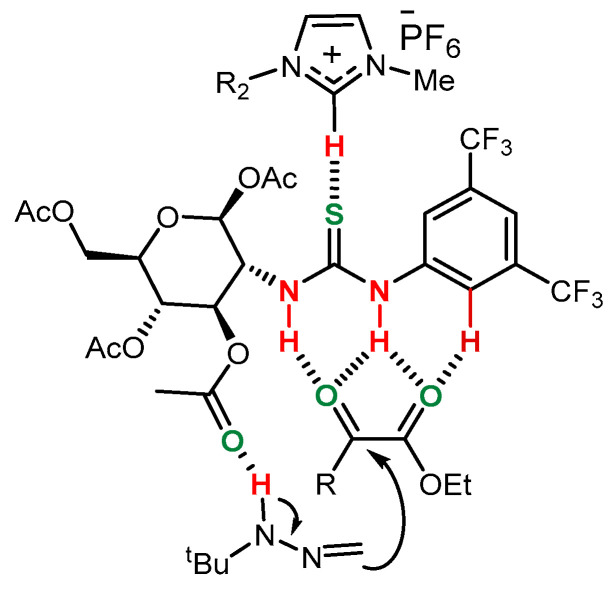
Proposed model for the enhancement of the (thio)urea catalyst **II** acidity by the interaction of imidazolium cation with the (thio)urea moiety.

There is not a clear explanation about the influence of RTILs in organocatalyzed reactions, despite the fact that many examples have been described in which these compounds have performed a beneficial effect in catalytic systems. In our case, a plausible explanation of the observed positive effect on catalyst performance is that the RTIL increased the acidity of the (thio)urea moiety by a hydrogen bonding interaction, as proposed in Figure 1 [30,37,38,39]. In this way, the ionic liquid (IL) strengthened the interaction between the catalyst and the α-keto ester. It must be pointed out that in toluene, the IL ion pair is in tight proximity, so the ionic liquid should be considered as a single zwitterionic specie rather than a pair of free counter ions. On the other hand, the poor Lewis basic character (or coordination ability) of the BF_4_^−^ or PF_6_^−^ counteranions enhances the relatively high acidity of the imidazolium cation and its availability to interact with the catalyst [40,41]. Moreover, such interactions will also have a beneficial effect by avoiding catalyst self-aggregation and, consequently, increasing the solubility of the catalyst [33] at lower temperatures. This assumption is in line with the results obtained with catalysts **II** and **III**, showing that the thiourea moiety, being essentially more acidic than the urea analogue, provided a higher catalytic activity. Finally, this rationale is also in agreement with the fact that the IL did not significantly interfere/modify the chiral recognition mode, since the enantiomeric excesses were quite similar to those obtained in the absence of RTIL (for instance, see entries 11–15, experiments conducted at the same temperature (–45 °C)).

**Figure 2:**
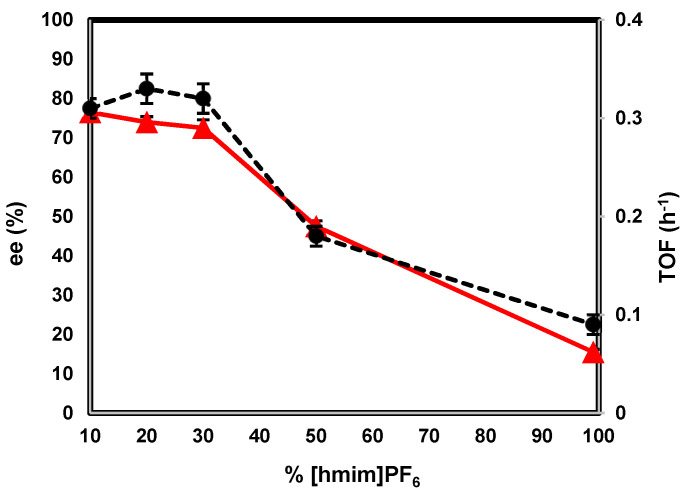
Effect of the [hmim]PF_6_ amount in the TOF (●) and the optical purity (▲) of (*S*)-**3a** for the thiourea **II** organocatalyzed addition of **1** to ethyl 2-oxo-4-phenylbutyrate (**2a**) catalyzed in toluene at −45 °C. Reported values are the mean of two replicates, with the uncertainties compounded from the TOF and optical purity data.

The effect of some reaction parameters on the enantiomeric excess of the products (addition of **1** to **2a** catalyzed by **II** in mixtures toluene/[hmim]PF_6_) were analyzed. Thus, the synthesis of (*S*)-**3a** was carried out using different volumetric proportions of the ionic liquid, as shown in Figure 2. The reactions could be performed with good results in terms of activity and selectivity using up to 30 vol% of [hmim]PF_6_, while higher RTIL proportions led to an important drop in both the conversion toward the (*S*)-azomethyl alcohol and its enantiomeric purity. In pure RTIL, the reaction still proceeded, but neither conversion nor selectivity were satisfactory. Different groups have studied the dependence between the RTIL proportion and the reaction outcome, being observed herein a similar trend to what was previously reported by Harper et al. [42] with a more pronounced effect of [hmim]PF_6_ between 10–30 vol%. The effect of substrate concentration was also analyzed in the preparation of (*S*)-**3a** while keeping constant the amount of catalyst. Reactions were typically performed at keto ester concentrations of 0.6 M. When increasing the substrate concentration up to 1.0 M, a 31% conversion was obtained after 24 h (0.22 h^−1^ TOF), whereas there was a slight loss in the enantioselectivity, as the product **3a** was obtained with 63% ee. The use of 0.3 M of **2a** also afforded (*S*)-**3a** with a lower TOF (0.14 h^−1^) with respect to that observed at 0.6 M. A 65% conversion was measured after 24 h with the same optical purity. Reactions were also performed at lower substrate concentrations (0.1 M and 0.05 M), which obtained lower TOF values (0.12 and 0.11 h^−1^, respectively), whereas the optical purities were slightly higher than at 0.6 M concentration, as (*S*)-**3a** was recovered with 78% ee for both concentrations. Overall, an α-keto ester concentration of 0.6 M seemed to be the best for this organocatalyzed process.

Once selecting both [bmim]BF_4_ and [hmim]PF_6_ as the best cosolvents/additives for the organocatalyzed additions, we examined the synthesis of the isopropyl derivative (*S*)-**3b** (Table 1, entries 18–21). This compound was obtained with 98% conversion and 58% ee when employing toluene at −15 °C [29]. When the reaction was carried out at −45 °C in the same reaction medium (entry 18), (*S*)-**3b** was recovered with 29% conversion and 62% ee. The presence of 10 vol% of either of these two selected RTILs at −45 °C allowed obtaining the final product with a slightly higher optical purity (66–67% ee). The use of [hmim]PF_6_ led to a higher conversion (75% after 24 h, entry 20), and this RTIL was employed for the other substrates **2c**–**g**. Addition of **1** to **2b** was also catalyzed by urea **III**. In the presence of 10 vol% [hmim]PF_6_, (*S*)-**3b** was obtained with 61% conversion and 52% ee, as shown in entry 22. 

**Scheme 2:**
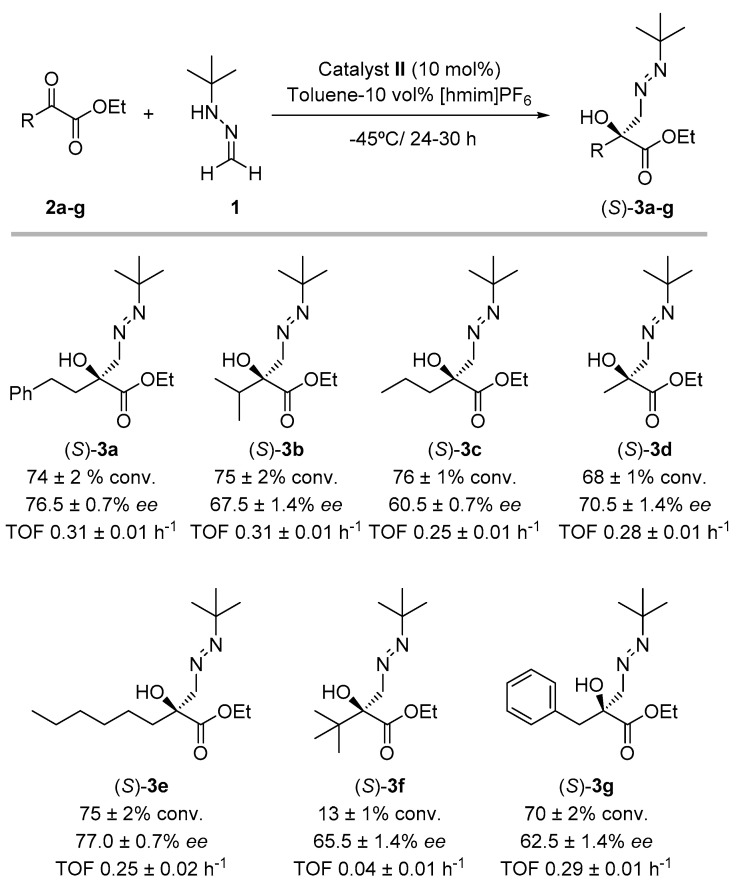
Substrate scope for the enantioselective addition of hydrazone **1** to different aliphatic α-ketoesters catalyzed by **II** in toluene containing a 10 vol% of [hmim]PF_6_ (4.6% *X*_[hmim]PF6_). Reactions were performed at −45 °C for 30 h, except for **2a** (24 h). Reported values are the mean of two replicates, with the uncertainties reported as half of the range.

The best conditions found for the enantioselective preparation of both (*S*)-**3a** and (*S*)-**3b**, using [hmim]PF_6_ at 10 vol% in toluene (4.6% molar fraction of this RTIL) and −45 °C, were extended to other substrates (Scheme 2). Thus, the addition of hydrazone **1** to the *n*-propyl keto ester **2c** afforded the azomethyl alcohol (*S*)-**3c** with 61% ee (0.25 h^−1^) in toluene/RTIL, which represents a 16% increase in the optical purity value with respect to the process in toluene at −15 °C. The methyl derivative (*S*)-**3d** was obtained with a 71% ee and a 68% conversion (0.28 h^−1^) after 24 h in the reaction catalyzed by **II** at –45 °C in the presence of 10 vol% [hmim]PF_6_. The presence of a longer alkyl chain (*n*-hexyl) in the α-keto ester (**2e**) allowed obtaining the final azomethyl alcohol with 75% conversion after 24 h at –45 °C and 77% ee (0.25 h^−1^), which represents a 14% increase in the optical purity regarding the reaction in toluene. A bulky alkyl substrate as **2f** was also tested. The reaction in toluene led to a low conversion even at 0 °C. When the reaction was performed at –30 °C using [hmim]PF_6_, (*S*)-**3f** was obtained with a very low conversion after 30 h (13%, 0.04 h^−1^), while for this compound the presence of the ionic liquid did not improve the enantiomeric excess. This low productivity may be due to steric clash between the *^t^*Bu group of the substrate and the organocatalyst, thus establishing a loose interaction and precluding a proper activation. Noteworthy, the *^i^*Pr-derivative **2b** (75% conversion, 67% ee (0.31 h^−1^)) and the *^t^*Bu-containing **2f** (13% conversion, 66% ee (0.04 h^−1^)) only differed in a methyl group. This exemplifies that both substrates have the same substrate–catalyst binding mode (almost identical enantioselectivity) but a different extent of activation. The novel reaction medium was extended to the benzylic derivative **2g**, for which a 63% ee was observed when employing 10 vol% [hmim]PF_6_, while a 70% conversion was achieved after 24 h (0.29 h^−1^).

**Scheme 3:**
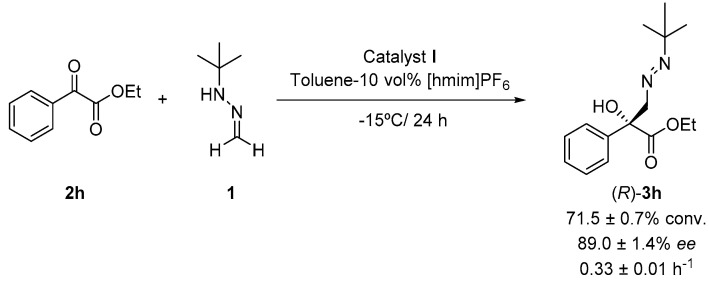
Enantioselective addition of hydrazone **1** to ethyl benzoyformate (**2h**) catalyzed by bis-urea **I** in toluene containing a 10 vol% of [hmim]PF_6_. Reported values are the mean of two replicates, with the uncertainties reported as half of the range.

In order to spread the benefits of the optimized reaction medium to other hydrogen-bonding catalysts, we analyzed the addition of hydrazone **1** to ethyl benzoylformate **2h** catalyzed by (*R*)-BINAM-derived bis-urea **I** (Scheme 3). The reaction performed in toluene at −15 °C led to a 53% conversion of the azomethyl alcohol (*R*)-ethyl 3-(*tert*-butyldiazenyl)-2-hydroxy-2-phenylpropanoate (**3h**) after 24 h (0.22 h^−1^), while the addition of the IL increased the conversion up to 71% (0.33 h^−1^). For both reactions, the optical purity of the final product was the same (89% ee). 

## 3. Materials and Methods

### 3.1. General Materials and Methods

NMR spectra were recorded by ^1^H NMR (300 or 400 MHz) and ^13^C NMR (75 or 100 MHz) with the solvent peak used as the internal reference (7.26 and 77.0 ppm for ^1^H and ^13^C, respectively) on a Bruker AC-300-DPX. Column chromatography was performed on silica gel (Merck Kieselgel 60, Merck, Kenilworth, United States). Analytical TLC was performed on aluminum backed plates (1.5 × 5 cm) precoated (0.25 mm) with silica gel (Merck, Silica Gel 60 F_254_, Merck, Kenilworth, United States). The compounds were visualized by exposure to UV light or by dipping the plates into solutions of KMnO_4_ or vanillin, followed by heating. 

Analytical grade solvents and commercially available reagents supplied by Sigma-Aldrich (Steinheim, Germany) and TCI Europe (Zwijndrecht, Belgium) were used without further purification. Formaldehyde *tert*-butyl hydrazone (**1**) [43], organocatalysts **I** [30], **II**, and **III [29]**, and noncommercially available α-keto esters **2c** and **2e** [44,45] were prepared according to literature procedures. All the synthesized compounds presented the same physical and spectroscopical properties as those previously described. 

### 3.2. Typical Procedure for the Hetero-Ene Reaction Catalyzed by (Thio)Ureas **I**–**III**

Formaldehyde *tert*-butyl hydrazone **1** (134 μL, 1.2 mmol) was added to a solution of α-keto ester **2a**–**h** (0.6 mmol) and catalyst **I**–**III** (0.06 mmol) in the corresponding toluene/RTIL mixture (total volume 0.6 mL) at the selected temperatures. Reactions were stirred for the established times (TLC monitoring). The solvent was removed under reduced pressure and the conversions were determined by ^1^H-NMR in CDCl_3_. For some selected reactions, flash chromatography purifications were performed in different toluene/EtOAc mixtures to measure the isolated yield of the corresponding optically active azomethyl alcohols (*S*)-**3a**–**g** and (*R*)-**3h** (see Supplementary Materials). Physical and spectroscopical properties of the azomethyl compounds **3a**–**h** matched those previously reported [29,30]. The optical purity of compounds (*S*)-**3a**, (*S*)-**3c**, (*S*)-**3g**, and (*R*)-**3h** was directly determined by HPLC on chiral stationary phases, while (*S*)-**3b** and (*S*)-**3d**–**f** were previously converted into their corresponding azoxymethyl alcohols by treatment with magnesium monoperoxyphthalate (MMPP) in methanol (for additional details see Supplementary Materials, Table S1).

## 4. Conclusions

The addition of certain imidazole-based ionic liquids ([bmim]BF_4_ and [hmim]PF_6_) in the thiourea-organocatalyzed addition of formaldehyde *tert*-butyl hydrazine (**1**) to different aliphatic α-keto esters (**2a**–**h**) had a positive effect in the catalyst activity. Thus, reactions can be conducted at lower temperatures, which allow obtaining the corresponding chiral azomethyl alcohols with moderate to good conversions and optical purities higher than those obtained in the absence of ionic liquids for most of the substrates tested, making it possible to attain enantiomeric excesses around 70–77% and up to a 3-fold TOF increase. To the best of our knowledge, this is one of the few examples of the positive effect of engineering the reaction of medium properties by adding ionic liquids in hydrogen bond organocatalysis. RTILs may find application in improving the performance of highly selective but “lazy” catalysts or enabling catalysis in otherwise nonproductive extreme temperature regimes.

## Figures and Tables

**Table 1:** Organocatalyzed addition of formaldehyde *tert*-butylhydrazone (FTBH) (**1**) to ethyl 2-oxo-4-phenylbutanoate (**2a**) and ethyl 3-methyl-2-oxobutanoate (**2b**) catalyzed by (thio)ureas **II** and **III** in toluene/RTIL (10 vol%) ^1^.

Entry	Ketone	RTIL	Cat.	*X*_RTIL_ (%)	T (°C)	Time (h)	Conv. (%) ^2^	ee (%) ^3^	TOF (h^−1^)
entry 1	**2a**	None	**II**	0	−15	16	68 ± 1	63.5 ± 0.7	0.43 ± 0.01
entry 2	**2a**	[bmim]MeSO_4_	**II**	5.4	−15	7	60 ± 1	13.5 ± 2.1	0.85 ± 0.01
entry 3	**2a**	[bmim]BF_4_	**II**	5.9	−15	7	77 ± 4	63.3 ± 0.6	1.10 ± 0.05
entry 4	**2a**	[bmim]PF_6_	**II**	5.3	−15	7	71 ± 1	56.5 ± 2.1	1.01 ± 0.02
entry 5	**2a**	[hmim]PF_6_	**II**	4.6	−15	7	80 ± 1	64.6 ± 1.1	1.15 ± 0.01
entry 6	**2a**	[hmim]NTf_2_	**II**	3.5	−15	7	48 ± 1	50.0 ± 1.4	0.59 ± 0.1
entry 7	**2a**	[bmp]BF_4_	**II**	5.6	−15	7	45 ± 1	54.5 ± 2.1	0.64 ± 0.02
entry 8	**2a**	None	**II**	0	−30	22	29 ± 2	67.5 ± 0.7	0.13 ± 0.01
entry 9	**2a**	[bmim]BF_4_	**II**	5.9	−30	22	83 ± 1	69.5 ± 0.7	0.38 ± 0.01
entry 10	**2a**	[hmim]PF_6_	**II**	4.6	−30	22	89 ± 2	70.5 ± 0.7	0.40 ± 0.02
entry 11	**2a**	None	**II**	0	−45	24	21 ± 1	71.5 ± 2.1	0.09 ± 0.01
entry 12	**2a**	[bmim]BF_4_	**II**	5.9	−45	24	68 ± 2	74.7 ± 0.6	0.28 ± 0.01
entry 13	**2a**	[bmim]BF_4_	**III**	5.9	−45	24	57 ± 2	66.5 ± 0.7	0.24 ± 0.01
entry 14	**2a**	[hmim]PF_6_	**II**	4.6	−45	24	74 ± 2	76.5 ± 0.7	0.31 ± 0.01
entry 15	**2a**	[hmim]PF_6_	**III**	4.6	−45	24	69 ± 1	66.5 ± 0.7	0.29 ± 0.01
entry 16	**2a**	None	**II**	0	−60	72	16 ± 1	72.0 ± 1.4	0.03 ± 0.01
entry 17	**2a**	[hmim]PF_6_	**II**	4.6	−60	72	34 ± 3	74.6 ± 1.5	0.06 ± 0.01
entry 18	**2b**	None	**II**	0	−45	24	29 ± 2	62.0 ± 1.4	0.12 ± 0.01
entry 19	**2b**	[bmim]BF_4_	**II**	5.9	−45	24	61 ± 2	66.3 ± 1.1	0.26 ± 0.01
entry 20	**2b**	[hmim]PF_6_	**II**	4.6	−45	24	75 ± 2	67.3 ± 0.6	0.31 ± 0.01
entry 21	**2b**	[hmim]PF_6_	**III**	4.6	−45	30	61 ± 2	51.5 ± 2.1	0.25 ± 0.02

^1^ Reported values are the mean of two or three replicates at each condition, with the uncertainties compounded from the conversion, optical purity, and turnover frequency (TOF) data. ^2^ Determined by ^1^H-NMR in CDCl_3_. ^3^ Determined directly by HPLC for compound **3a** while compound **3b** was converted into its azoxymethyl derivative before HPLC analysis. *X*_RTIL_: Molar fraction of the RTIL, represents the moles of RTIL divided by the total moles of solution (RTIL + toluene).

## Data Availability

Data is contained within the article or supplementary material.

## Supplementary Materials

The following are available online, Experimental procedures and Table S1: HPLC conditions for the determination of the enantiomeric excess of azomethyl alcohols **3a**–**h**.
