# Supplementary material for: Room Temperature Ionic Liquids in Asymmetric Hetero-Ene Type Reactions: Improving Organocatalyst Performance at Lower Temperatures
Source: Molecules. 2021 Jan 12;26(2):355. doi: 10.3390/molecules26020355 (PMC7827838; doi:10.3390/molecules26020355)
Supplement: Supplementary file 1 [file molecules-26-00355-s001.pdf]

molecules

Supporting Information

Room temperature ionic liquids in asymmetric hetero-ene type reactions: Improving organocatalyst performance at lower temperatures

Fabricio R. Bisogno,[1] Rosario Fernández,[2] José M. Lassaletta[3] and Gonzalo de Gonzalo[2,*]

[1] Facultad de Ciencias Químicas, Instituto de Investigaciones en Físico-Química de Córdoba, Universidad Nacional de Córdoba, Haya de la Torre y Medina Allende, Ciudad Universitaria, 5000, Córdoba, Argentina.

[2] Departamento de Química Orgánica, Universidad de Sevilla, c/ Profesor García González 1, 41012, Sevilla, Spain.

[3] Instituto de Investigaciones Químicas (CSIC-US) and Centro de Innovación en Química Avanzada (ORFEO-CINQA), Avda. Américo Vespucio 49, 41092, Sevilla, Spain.

1. Experimental procedures……………………………………………………………………2
2. HPLC analyses………………………………………………………………………………4
3. References…………………………………………………………………………………...5

1. Experimental procedures.

1.1. Organocatalyzed addition of hydrazone 1 to α-ketoesters 2a-f catalyzed by thiourea III in presence of 10% v/v [hmim]PF_6.

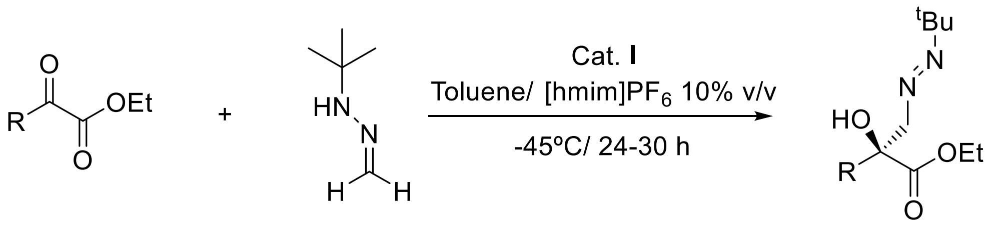

2a-g **1** (*S*)-**3a-g**

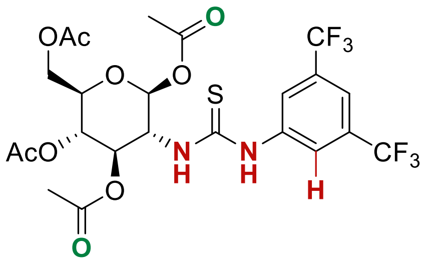

III

Formaldehyde *tert*-butyl hydrazone **1** (134 µL, 1.2 mmol) was added to a solution of α-ketoester **2a-g** (0.6 mmol) and catalyst **I** (0.06 mmol) in 0.6 mL of the mixture toluene/[hmim]PF_6 9:1 at -45 ℃. Reactions were stirred for 24-30 hours and solvent was removed under reduced pressure. The crude mixtures were analyzed by ^{1}H-NMR in $CDCl_3$ in order to determine the conversion of the reactions: 73% for **2a**, 77% for **2b**, 75% for **2c**, 67% for **2d**, 74% for **2e**, 13% for **2f** and 69% for **2g**. Flash chromatography was then performed using as eluent toluene/EtOAc 8:1 mixture for compounds **3a-f** and toluene/EtOAc 6:1 for compound **2g** to afford the corresponding optically active azomethyl alcohols (*S*)-**3a** with 60% yield (110.2 mg), (*S*)-**3b** with 68% yield (99.5 mg), (*S*)-**3c** with 58% yield (84.9 mg), (*S*)-**3d** with 61% yield (79.0 mg), (*S*)-**3e** with 65% yield (111.5 mg), (*S*)-**3f** with 8% yield (12.4 mg) and (*S*)-**3g** with 62% yield (108.6 mg). Physical and spectroscopical properties of compounds **3a-g** are in accord with those reported [1]. The optical purity of compounds (S)-**3a**, (*S*)-**3c** and (*S*)-**3g** was directly determined by HPLC on chiral stationary phases, while (*S*)-**3b**, (*S*)-**3d-f** were previously converted into their corresponding azoxymethyl alcohols by treatment with magnesium monoperoxyphthalate (MMPP) in methanol.

1.2.Organocatalyzed addition of hydrazone 1 to ethyl benzoylformate (2h) catalyzed by I.

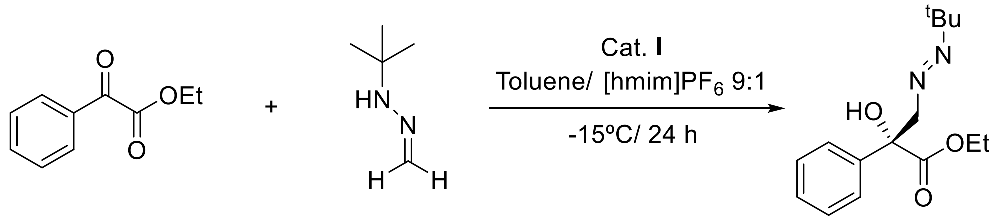

2h **1** (*R*)-**3h**

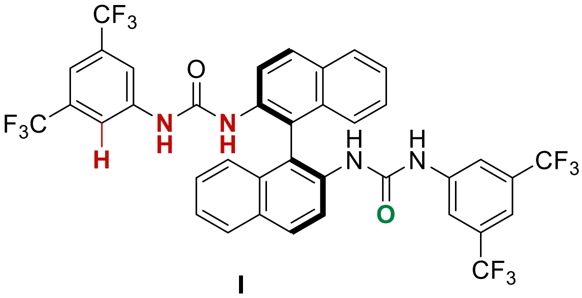

I

Formaldehyde *tert*-butyl hydrazone **1** (134 µL, 1.2 mmol) was added to a solution of ethyl benzoylfomate **2h** (0.6 mmol) and catalyst **I** (0.06 mmol) in 0.6 mL of the mixture toluene/[hmim]PF_6 9:1 at -15 ℃. Reactions were stirred for 24 hours and solvent was removed under reduced pressure. The crude mixture was analysed by ^{1}H-NMR in $CDCl_3$ in order to determine the conversion of the reaction (71%). Flash chromatography was then performed using as eluent toluene/EtOAc 8:1 mixture to afford the corresponding optically active azomethyl alcohol (*R*)-**3h** with 62% yield (103.4 mg), whose physical and chemical properties are in accord with those reported [2]. The optical purity of (*R*)-**3h** (89% *ee*) was determined directly by HPLC or chiral stationary phase.

3. HPLC analyses

In order to determine the enantiomeric excesses of azomethyl alcohols (*S*)-**3b** and (*S*)-**3d-f**, these compounds have to be converted first in their azoxymethyl derivatives following the general procedure: (*S*)-**3b**,**d-f** (0.2 mmol) were dissolved in MeOH (2 mL), cooled to 0 ℃, and MMPP (0.5 g, 5 equiv.) was added. The reaction mixture was stirred until the consumption of the azomethyl alcohol **3** (TLC monitoring toluene/EtOAc 6:1, 2–3 h.), diluted with H_2O (5 mL) and extracted with CH_2Cl_2 (3 × 5 mL). The organic phases were dried over Na_2SO_4 and concentrated in *vacuo*. The resulting residue was purified by flash chromatography (toluene/EtOAc 6:1) to afford the corresponding azoxymethyl alcohols with yields around 75-90%.

HPLC analyses were performed on a Waters 2695 Instrument equipped with a Waters 996 Photodiode Array Detector. The following columns were employed for the determination of the optical purities of azomethyl alcohols (*S*)-**3a-g** and (*R*)-**3h**: Column A: Chiralpak AD-H (0.46 cm x 25 cm, Daicel) and column B: Chiralpak OJ-H (0.46 cm x 25 cm, Daicel):

Table S.1. Determination of enantiomeric excess values by HPLC.

Product	Column	Flow rate [mL min^{-1}]	T [℃]	Eluent[a]	Retention time [min]
**3a**	A	1.0	30	*n*-hexane/IPA 98:2	7.8 (*R*); 8.9 (*S*)
**3b**	A	1.0	30	*n*-hexane/IPA 98:2	9.9 (*R*); 10.5 (*S*)
**3c**	A	1.0	30	*n*-hexane/IPA 98:2	5.6 (*R*); 6.7 (*S*)
**3d**	B	1.0	30	*n*-hexane/IPA 98:2	11.5 (*S*); 13.5 (*R*)
**3e**	A	1.0	30	*n*-hexane/IPA 98:2	4.3 (*R*); 4.9 (*S*)
**3f**	A	1.0	30	*n*-hexane/IPA 9:1	4.1 (*R*); 4.6 (*S*)
**3g**	B	1.0	30	*n*-hexane/IPA 98:2	13.5 (*S*); 14.9 (*R*)
**3h**	A	1.0	30	*n*-hexane/IPA 98:2	12.7 (*R*); 20.8 (*S*)

[a] All the experiments were performed with isocratic eluent.

4. References

[1] Carmona, J. A.; de Gonzalo, G.; Serrano, I.; Crespo-Peña, A. M.; Simek, M.; Monge, D.; Fernández, R.; Lassaletta, J. M. Asymmetric organocatalytic synthesis of tertiary azomethyl alcohols: key intermediates towards azoxy compounds and α-hydroxy-β-amino esters. *Org. Biomol. Chem.* **2017**, *15*, 2993–3005.

[2] Crespo-Peña, A.; Monge, D.; Martín-Zamora, E.; Álvarez, E.; Fernández, R.; Lassaletta, J. M. Asymmetric formal carbonyl-ene reactions of formaldehyde *tert*-butyl hydrazone with α-keto esters: Dual activation by Bis-urea catalysts. *J. Am. Chem. Soc.* **2012**, *134*, 12912–12915.
